# Reinforcement Learning or Active Inference?

**DOI:** 10.1371/journal.pone.0006421

**Published:** 2009-07-29

**Authors:** Karl J. Friston, Jean Daunizeau, Stefan J. Kiebel

**Affiliations:** The Wellcome Trust Centre for Neuroimaging, University College London, London, United Kingdom; Indiana University, United States of America

## Abstract

This paper questions the need for reinforcement learning or control theory when optimising behaviour. We show that it is fairly simple to teach an agent complicated and adaptive behaviours using a free-energy formulation of perception. In this formulation, agents adjust their internal states and sampling of the environment to minimize their free-energy. Such agents learn causal structure in the environment and sample it in an adaptive and self-supervised fashion. This results in behavioural policies that reproduce those optimised by reinforcement learning and dynamic programming. Critically, we do not need to invoke the notion of reward, value or utility. We illustrate these points by solving a benchmark problem in dynamic programming; namely the mountain-car problem, using active perception or inference under the free-energy principle. The ensuing proof-of-concept may be important because the free-energy formulation furnishes a unified account of both action and perception and may speak to a reappraisal of the role of dopamine in the brain.

## Introduction

Traditionally, the optimization of an agent's behaviour is formulated as maximizing value or expected reward or utility or [1]–[8]. This is seen in cognitive psychology, through the use of reinforcement learning models like the Rescorla-Wagner model [1]; in computational neuroscience and machine-learning as variants of dynamic programming, such as temporal difference learning [2]–[7] and in economics, as expected utility theory [8]. In these treatments, the problem of optimizing behaviour is reduced to optimizing expected reward or utility (or conversely minimizing expected loss or cost). Effectively, this prescribes an optimal policy in terms of the reward that would be expected by pursuing that policy. Our work suggests that this formulation may represent a slight misdirection in explaining adaptive behaviour. In this paper, we specify an optimal policy in terms of the probability distribution of desired states and ask if this is a simpler and more flexible approach. Under this specification, optimum behaviour emerges in agents that conform to a free-energy principle, which provides a principled basis for understanding both action and perception [9], [10]. In what follows, we review the free-energy principle, show how it can be used to solve the mountain-car problem [11] and conclude by considering the implications for the brain and behaviour.

## Methods

### The free-energy principle

We start with the premise that adaptive agents or phenotypes must occupy a limited repertoire of states. See Friston et al [9] for a detailed discussion: In brief, for a phenotype to exist it must possess defining characteristics or *traits*; both in terms of its morphology and exchange with the environment. These traits essentially limit the agent to a bounded region in the space of all states it could be in. Once outside these bounds, it ceases to possess that trait (cf, a fish out of water). This speaks to self-organised autopoietic interactions with the world that ensure phenotypic bounds are never transgressed (cf, [12]). In what follows, we formalise this notion in terms of the entropy or average surprise associated with a probability distribution on an agent's state-space. The basic idea is that adaptive agents occupy a compact part of this space and therefore minimise the average surprise associated with finding itself in unlikely states (cf, a fish out of water - sic). Starting with this defining attribute of adaptive agents, we will look at how agents might minimise surprise and then consider what this entails, in terms of their action and perception.

The free-energy principle starts with the notion of an ensemble density 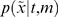 on the generalised states [13], 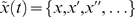 an agent, *m* can find itself in. Generalised states cover position, velocity, acceleration, jerk and so on. We assume these states evolve according to some complicated equations of motion; 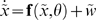, where *w* are random fluctuations, whose amplitude is controlled by 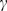. Here, 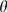 are parameters of a nonlinear function 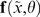, encoding environmental dynamics. Collectively, causes 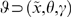 are all the environmental quantities that affect the agent, such as forces, concentrations, rate constants and noise levels. Under these assumptions, the evolution of the ensemble density is given by the Fokker-Planck equation
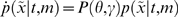
(1)


Where 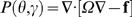 is the Fokker-Planck operator and 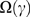 is a diffusion tensor corresponding to half the covariance of 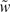. The Fokker-Planck operator plays the role of a probability transition matrix and determines the ultimate distribution of states that agents will be found in. The solution to Equation 1, for which 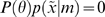, is the equilibrium density (i.e., when the density stops changing) and depends only on the parameters controlling motion and the amplitude of the random fluctuations. This equilibrium density 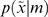 can be regarded as the probability of finding an agent in a particular state, when observed on multiple occasions or, equivalently, the density of a large ensemble of agents at equilibrium with their environment. Critically, for an agent to exist, the equilibrium density should have low entropy. As noted above, this ensures that agents occupy a limited repertoire of states because a low entropy density has most of its mass in a small part of its support. This places an important constraint on the states sampled by an agent; it means agents must somehow minimise their equilibrium entropy and counter the dispersive effects of random or deterministic forces. In short, biological agents must resist a natural tendency to disorder; but how do they do this?

### Active agents

At this point, we introduce the notion of active agents [14] that sense a subset of states (with sensory organs) and can change others (with effector organs). We can quantify this exchange with the environment with sensory, 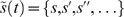 and action or control signals, *a*(*t*). We will describe sensory sampling (e.g., retinotopic encoding) as a probabilistic mapping, 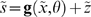, where *z* is sensory noise. Control (e.g., saccadic eye-movements) can be represented by treating action as a state; 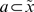; which we will call hidden states from now on because they are not sensed directly. From the point of view of reinforcement learning and optimum control theory, action depends on sensory signals, where this dependency constitutes a policy, 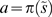.

Under a sensory mapping, the equilibrium entropy is bounded by the entropy of sensory signals minus a sensory transfer term
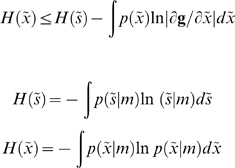
(2)with equality in the absence of sensory noise. This means it is sufficient to minimise the terms on the right to minimise the equilibrium entropy of hidden states. The second term depends on the sensitivity of sensory inputs to changes in the agent's states, where 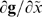 is the derivative of the sensory mapping with respect to generalised hidden states. Minimising this term maximises the mutual information between hidden states and sensory signals. This recapitulates the principle of maximum information transfer [15], which has been very useful in understanding things like receptive fields [16]. Put simply, sensory channels should match the dynamic range of states they sample (e.g., the spectral sensitivity profile of photoreceptors and the spectrum of ambient light).

In the present context, the interesting term is the sensory entropy; 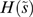, which can be minimised through action because sensory signals depend upon hidden states, which include action. The argument here is that it is necessary but not sufficient to minimise the entropy of the sensory signals. To ensure the entropy of the hidden states *per se* is minimised one has to assume the agent is equipped with (and uses) its sensory apparatus to maximise information transfer. We will assume this is assured through natural selection and focus on the minimisation of sensory entropy:

Crucially, because the density on sensory signals is at equilibrium, it can be interpreted as the proportion of time each agent entertains these signals (this is called the sojourn time). This ergodic argument [17] means that the ensemble entropy is the long-term average or path integral of 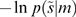 experienced by any particular agent:



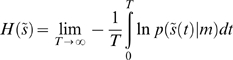
(3)


In other words, active agents minimise 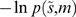 over time (by the fundamental lemma of the calculus of variations). This quantity is known as self-information or *surprise* in information theory (and as the negative log-evidence in statistics). When friends and colleagues first come across this conclusion, they invariably respond with; “but that means I should just close my eyes or head for a dark room and stay there”. In one sense this is absolutely right; and is a nice description of going to bed. However, this can only be sustained for a limited amount of time, because the world does not support, in the language of dynamical systems, stable fixed-point attractors. At some point you will experience surprising states (e.g., dehydration or hypoglycaemia). More formally, itinerant dynamics in the environment preclude simple solutions to avoiding surprise; the best one can do is to minimise surprise in the face of stochastic and chaotic sensory perturbations. In short, a necessary condition for an agent to exist is that it adopts a policy that minimizes surprise. However, there is a problem:

### The need for perception

The problem faced by real agents is that they cannot quantify surprise, which entails marginalizing over the unknown causes, 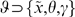 of sensory input
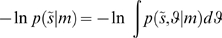
(4)


However, there is an alternative and elegant solution to minimizing surprise, which comes from theoretical physics [18] and machine learning [19], [20]. This involves minimizing a free-energy bound on surprise that can be evaluated (minimising the bound implicitly minimises surprise because the bound is always greater than surprise). This bound is induced by a recognition density; 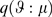, whose sufficient statistics 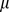 are, we assume, encoded by the internal states of the agent (e.g., neuronal activity or metabolite concentrations and connection strengths or rate-constants). The recognition density is a slightly mysterious construct because it is an *arbitrary* probability density specified by the internal states of the agent. Its role is to induce free-energy, which is a function of the internal states and sensory inputs. We will see below that when this density is optimised to minimise free-energy it becomes the conditional density on the causes of sensory data; in Bayesian inference this is known as the recognition density. In what follows, we try to summarise the key ideas behind a large body of work in statistics and machine learning referred to as *ensemble learning* or *variational Bayes*.

The free-energy can be defined as: (i) energy minus entropy, (ii) surprise plus the divergence between the recognition 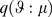 and conditional densities 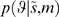 and (iii) complexity minus accuracy
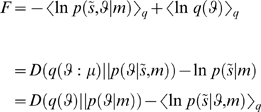
(5)


Here, 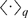
means the expected value or mean under the density *q* and 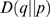 is the cross-entropy or Kullback-Leibler divergence between densities *q* and *p* (see Table 1). The alternative formulations in Equation 5 have some important implications: The first shows that free-energy can be evaluated by an agent; provided it has a probabilistic model of the environment. This generative model is usually expressed in terms of a likelihood and prior; 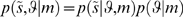. The second shows that minimizing the free-energy, by changing internal states, reduces the divergence between the recognition and posterior densities; rendering the recognition density an approximate conditional density. This corresponds to Bayesian inference on the causes of sensory signals and provides a principled account of perception; i.e., the Bayesian brain [21]–[31]. Critically, it also shows that free-energy is an upper bound on surprise because the divergence cannot be less than zero. In this paper, perception refers to inference on the causes of sensory input, not simply the measurement or sampling of sensory data. This inference rests on optimising the recognition density and implicit changes in the internal states of the agent.

**Table 1:** Glossary of mathematical symbols.

Variable	Short description
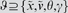	Environmental causes of sensory input
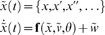	Generalised hidden-states of an agent. These are time-varying quantities that include all high-order temporal derivatives.
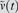	Generalised forces or causal states that act on hidden states
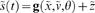	Generalised sensory states sampled by an agent
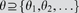	Parameters of 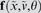 and 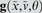
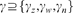	Parameters of the precisions of random fluctuations 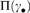
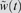	Generalised random fluctuations of the motion of hidden states
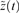	Generalised random fluctuations of sensory states
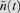	Generalised random fluctuations of causal states
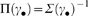	Precisions or inverse covariances of generalised random fluctuations
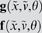	Sensory mapping and equations of motion generating sensory states
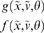	Sensory mapping and equations of motion used to model sensory states
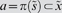	Action: a policy function of generalised sensory states; a hidden state that the agent can change
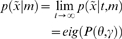	Equilibrium ensemble density; the density of an ensemble of agents at equilibrium with their environment. It is the principal eigensolution of the Fokker-Plank operator
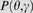	Fokker-Plank operator that is a function of fixed causes
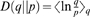	Kullback-Leibler divergence; also known as relative-entropy, cross-entropy or information gain
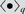	Expectation or mean under the density *q*
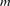	Model or agent; entailing the form of a generative model
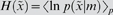	Entropy of generalised hidden states
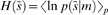	Entropy of generalised sensory states
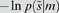	Surprise or self-information of generalised sensory states
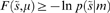	Free-energy bound on surprise
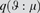	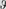 Recognition density on the causes
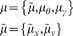	Conditional or posterior expectation of the causes 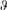 ; these are sufficient statistics of the recognition density
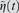	Prior expectation of generalised causal states
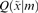	Desired equilibrium density
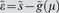	Generalised prediction error on sensory states

The third equality shows that free-energy can also be suppressed by action, through its vicarious effects on sensory signals. In short, the free-energy principle prescribes perception and an optimum policy
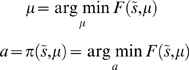
(6)


This policy reduces to sampling input that is expected under the recognition density (i.e., sampling selectively what one expects to see, so that accuracy is maximised; Equation 5). In other words, agents must necessarily (if implicitly) make inferences about the causes of their sensory signals and sample those that are consistent with those inferences. This is similar to the notion that “perception and behaviour can interact synergistically, via the environment” to optimise behaviour [32]. Furthermore, it echoes recent perspectives on sequence learning that “minimize deviations from the desired state, that is, to minimize disturbances of the homeostasis of the feedback loop”. See Wörgötter & Porr [33] for a fuller discussion.

At first glance, sampling the world to ensure it conforms to our expectations may seem to preclude exploration or sampling salient information. However, the minimisation in Equation 6 could use a stochastic search; sampling the sensorium randomly for a percept with low free-energy. Indeed, there is compelling evidence that our eye movements implement an optimal stochastic strategy [34]. This raises interesting questions about the role of stochastic schemes; from visual search to foraging. However, in this treatment, we will focus on gradient descent.

### Summary

In summary, the free-energy principle requires the internal states of an agent and its action to suppress free-energy. This corresponds to optimizing a probabilistic model of how sensations are caused, so that the ensuing predictions can guide active sampling of sensory data. The resulting interplay between action and perception (i.e., active inference) engenders a policy that ensures the agent's equilibrium density has low entropy. Put simply, if you search out things you expect, you will avoid surprises. It is interesting that the second law of thermodynamics (which applies only to closed systems) can be resisted by appealing to the more general tendency of (open) systems to reduce their free-energy [35], [36]. However, it is important not to confuse the free-energy here with thermodynamic free-energy in physics. Variational free-energy is an information theory measure that is a scalar function of sensory states or data and a probability density (the recognition density). This means thermodynamic arguments are replaced by arguments based on population dynamics (see above), when trying to understand why agents minimise their free-energy. A related, if abstract, treatment of self-organisation in non-equilibrium systems can be found in synergetics; where “patterns become functional because they consume in a most efficient manner the gradients which cause their evolution” [37]. Here, these gradients can be regarded as surprise. Finally, Distributed Adaptive Control [38] also relates closely to the free-energy formulation, because it addresses the optimisation of priors and provides an integrated solution to both the acquisition of state-space models and policies, without relying on reward or value signals: see [32] and [38].

### Active inference

To see how active inference works in practice, one must first define an environment and the agent's model of that environment. We will assume that both can be cast as dynamical systems with additive random effects. For the environment we have
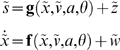
(7)which is modelled as
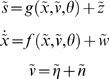
(8)


These stochastic differential equations describe how sensory inputs are generated as a function of hidden generalized states, 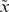 and exogenous forces, 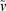 plus sensory noise, 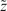. Note that we partitioned hidden states into hidden states and forces so that 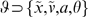. The hidden states evolve according to some equations of motion plus state noise, 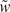. The use of generalised coordinates may seem a little redundant, in the sense that one might use a standard Langevin form for the stochastic differential equations above. However, we do not assume the random fluctuations are Weiner processes and allow for temporally correlated noise. This induces a finite variance on all higher derivatives of the fluctuations and necessitates the use of generalised coordinates. Although generalised coordinates may appear to complicate matters, they actually simplify inference greatly; see [13] and [39] for details.

Gaussian assumptions about the random fluctuations furnish a likelihood model; 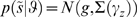 and, critically, priors on the dynamics, 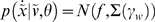. Here the inverse covariances or precisions 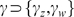 determine the amplitude and smoothness of the generalised fluctuations. Note that the true states depend on action, whereas the generative model has no notion of action; it just produces predictions that action tries to fulfil. Furthermore, the generative model contains a prior on the exogenous forces; 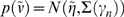. Here, 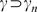 is the precision of the noise on the forces, 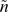 and is effectively a prior precision. It is important to appreciate that the equations actually generating data (Equation 7) and those employed by the generative model (Equation 8) do not have to be the same; indeed, it is this discrepancy that action tries to conceal. Given a specific form for the generative model the free-energy can now be optimised:

This optimisation obliges the agent to infer the states of the world and learn the unknown parameters responsible for its motion by optimising the sufficient statistics of its recognition density; i.e., perceptual inference and learning. This can be implemented in a biologically plausible fashion using a principle of stationary action as described in [39]. In brief, this scheme assumes a mean-field approximation; 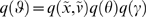 with Gaussian marginals, whose sufficient statistics are expectations and covariances. Under this Gaussian or Laplace assumption, it is sufficient to optimise the expectations, 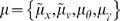 because they specify the covariances in closed form. Using these assumptions, we can formulate Equation 6 as a gradient descent that describes the dynamics of perceptual inference, learning and action: 
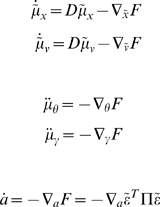
(9)


We now unpack these equations and what they mean. The top-two equations prescribe recognition dynamics on expected states of the world. The second terms of these equations are simply free-energy gradients. The first terms reflect the fact that we are working in generalised coordinates and ensure 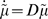 when free-energy is minimised and its gradient is zero (i.e., the motion of the expectations is the expected motion). Here, *D* is a derivative operator with identity matrices in the first leading diagonal. The solutions to the next pair of equations are the optimum parameters and precisions. Note that these are second-order differential equations because these expectations optimise a path-integral of free-energy; see [13] for details. The final equation describes action as a gradient descent on free-energy. Recall that the only way action can affect free-energy is through sensory signals. This means, under the Laplace assumption, action must suppress prediction error; 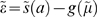 at the sensory level; where 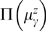 is the expected precision of sensory noise.

**Figure 1:**
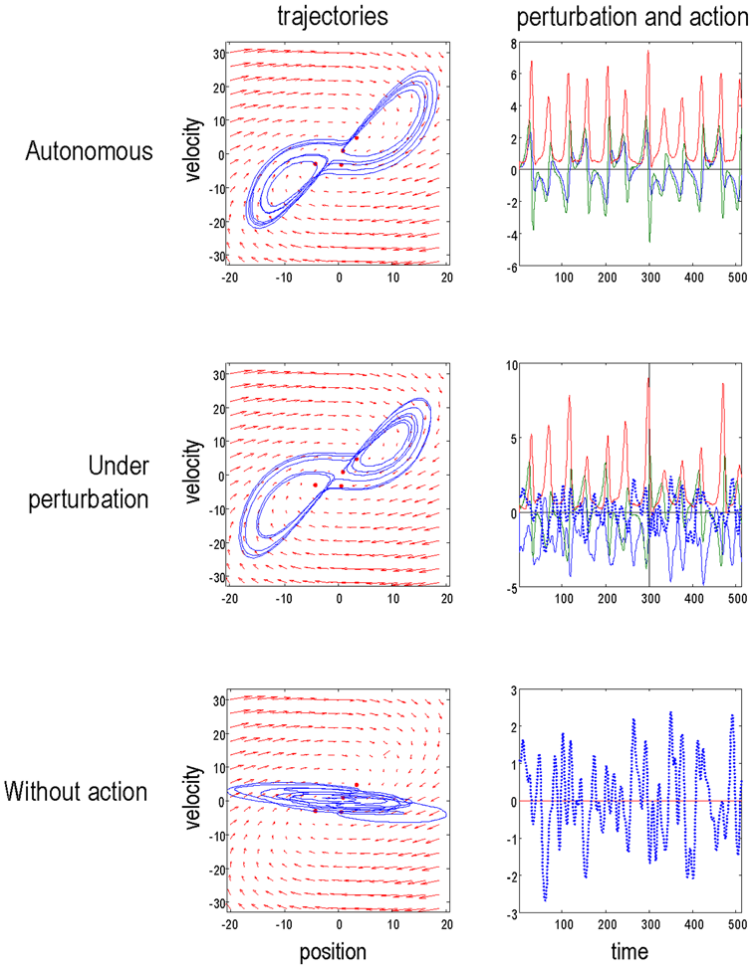
An agent that thinks it is a Lorenz attractor. This figure illustrates the behaviour of an agent whose trajectories are drawn to a Lorenz attractor. However, this is no ordinary attractor; the trajectories are driven purely by action (displayed as a function of time in the right panels). Action tries to suppress prediction errors on motion through this three dimensional state-space (blue lines in the left panels). These prediction errors are the difference between sensed and expected motion based on the agent's generative model; 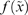 (red arrows: evaluated at 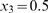). These prior expectations are based on a Lorentz attractor. The ensuing behaviour can be regarded as a form of chaos control. Critically, this autonomous behaviour is very resistant to random forces on the agent. This can be seen by comparing the top row (with no perturbations) with the middle row, where the first state has been perturbed with a smooth exogenous force (broken line). Note that action counters this perturbation and the ensuing trajectories are essentially unaffected. The bottom row shows exactly the same simulation but with action turned off. Here, the environmental forces cause the agents to precess randomly around the fixed point attractor of 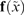. These simulations used a log-precision on the random fluctuations of 16.

Equation 9 embodies a nice convergence of action and perception; perception tries to suppress prediction error by adjusting expectations to furnish better predictions of signals, while action tries to fulfil these predictions by changing those signals. Figure 1 illustrates this scheme by showing the trajectory of an agent that thinks it is a strange attractor. We created this agent by making its generative model a Lorenz attractor:
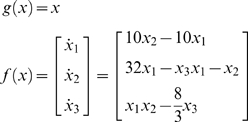
(10)


This means that the agent expects to move through the environment as if it was on a Lorenz attractor. Critically, the actual environment did not support any chaotic dynamics and, in the absence of action or exogenous forces, the states decay to zero
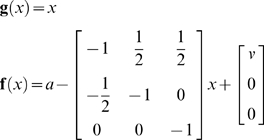
(11)


However, because we used a high log-precision of 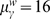, the agent's prior expectations about its motion created sufficiently strong prediction errors to support motion through state-space. As a result, the agent uses action to resolve this prediction error by moving itself. A log-precision of 16 means that the standard deviation is exp(−16/2) = 0.00034. This is quite small in relation to predicted motion, which means the predicted sensory states 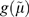 are dominated by the agent's prior expectations and the prediction error is explained away by action, as opposed to changes in conditional predictions.

To produce these simulations one has to integrate time-varying states in both the environment (Equation 7) and the agent (Equation 9) together, where hidden and expected states are coupled though action.
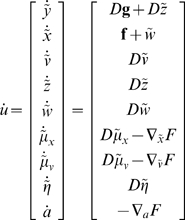
(12)


We use a local linearisation to update these states; 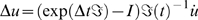 over time steps of 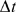, where the Jacobian 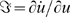 [40] and 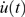 is given by A.1. This may look complicated but can be evaluated automatically using numerical derivatives. All the simulations in this paper use just one routine (**spm_ADEM.m**) and are available as part of the SPM software (http://www.fil.ion.ion.ucl.ac.uk/spm; **DEM_demo.m**).

Although an almost trivial example, this way of prescribing desired trajectories may have pragmatic applications in engineering and robotics [41], [42]. This is because the trajectories prescribed by active inference are remarkably robust to noise and exogenous perturbations (see Figure 1). In the next section, we return to the problem of specifying desired trajectories in terms of desired states, as opposed to trajectories *per se*.

## Results

The free-energy principle supposes that agents minimise the entropy of their equilibrium density. In ethology and evolutionary biology this may be sufficient, because the equilibria associated with phenotypes are defined through co-evolution and selection [43]–[44]: A phenotype exists because its ensemble density has low entropy; the entropy is low because the phenotype exists. It would be tautological to call these agents or their equilibria ‘optimal’. However, in the context of control theory, one may want to optimise policies to attain specific equilibria. For example, one might want to teach a robot to walk [41]. In what follows, we show how policies can be optimized under the free-energy principle, in relation to desired states that are prescribed by a density - 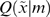. This is an equilibrium density one would like agents to exhibit; it allows one to specify the states the agent should work towards and maintain, under perturbations.

In brief, learning entails immersing an agent in a controlled environment that furnishes the desired equilibrium density. The agent learns the causal structure of this training environment and encodes it through perceptual learning as described above. This learning induces prior expectations that are retained when the agent is replaced in an uncontrolled or test environment. Because the agent samples the environment actively, it will seek out the desired sensory states that it has learned to expect. The result is an optimum policy that is robust to perturbations and constrained only by the agent's prior expectations that have been established during training. To create a controlled environment one can simply minimise the divergence between the uncontrolled equilibrium density and the desired density. We now illustrate this form of learning using a ubiquitous example from dynamic programming - the mountain-car problem.

### The mountain-car problem

**Figure 2:**
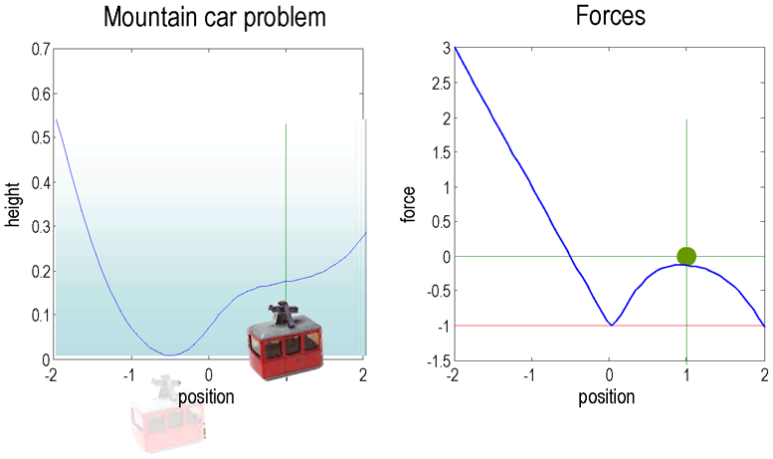
The mountain car problem. This is a schematic representation of the mountain car problem: Left: The landscape or potential energy function that defines the motion of the car. This has a minima at 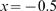. The mountain-car is shown at its uncontrolled stable position (transparent) and the desired parking position at the top of the hill on the right 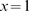. Right: Forces experienced by the mountain-car at different positions due to the slope of the hill (blue). Critically, at 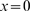 the force is minus one and cannot be exceeded by the cars engine, due to the squashing function applied to action.

In the mountain-car problem, one has to park a car on the top of a mountain (Figure 2). The car can be accelerated in a forward or reverse direction. The interesting problem here is that acceleration cannot overcome gravitational forces experienced during the ascent. This means that the only solution is to reverse up another hill and use momentum to carry it up the first. This represents an interesting problem, when considered in the state-space of position and velocity, 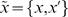; the agent has to move *away* from the desired location (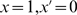) to attain its goal. This provides a metaphor for high-order conditioning, in which an agent must access goals vicariously, through sub-goals.

The mountain-car environment can be specified with the sensory mapping and equations of motions (where 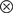 denotes the Kronecker tensor product)
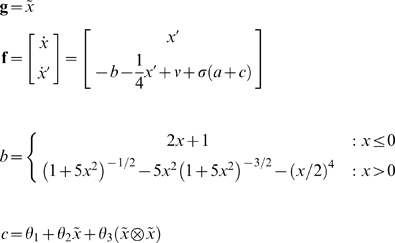
(13)


The first equality means the car has a (noisy) sense of its position and velocity. The second means that the forces on the car, 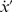 have four components: a gravitational force *b*, friction 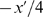, an exogenous force *v* and a force that is bounded by a squashing (logistic) function; 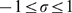. The latter force comprises action and a state-dependent control, *c*. Control is approximated here with a second-order polynomial expansion of any nonlinear function of the states, whose parameters are 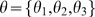. When 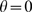 the environment is uncontrolled; otherwise the car experiences state-dependent forces that enable control.

To create a controlled environment that leads to an optimum equilibrium, we simply optimise the parameters to minimise the divergence between the equilibrium and desired densities; i.e.
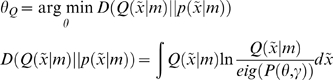
(14)


The equilibrium density is the eigensolution 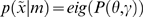 of the Fokker-Planck operator in Equation 1, which depends on the parameters and the precision of random fluctuations (we assumed these had a log-precision of 16). We find these eigensolutions by iterating Equation 1 until convergence to avoid inverting large matrices. The minimization above can use any nonlinear function minimization or optimization scheme; such as Nelder-Mead.

**Figure 3:**
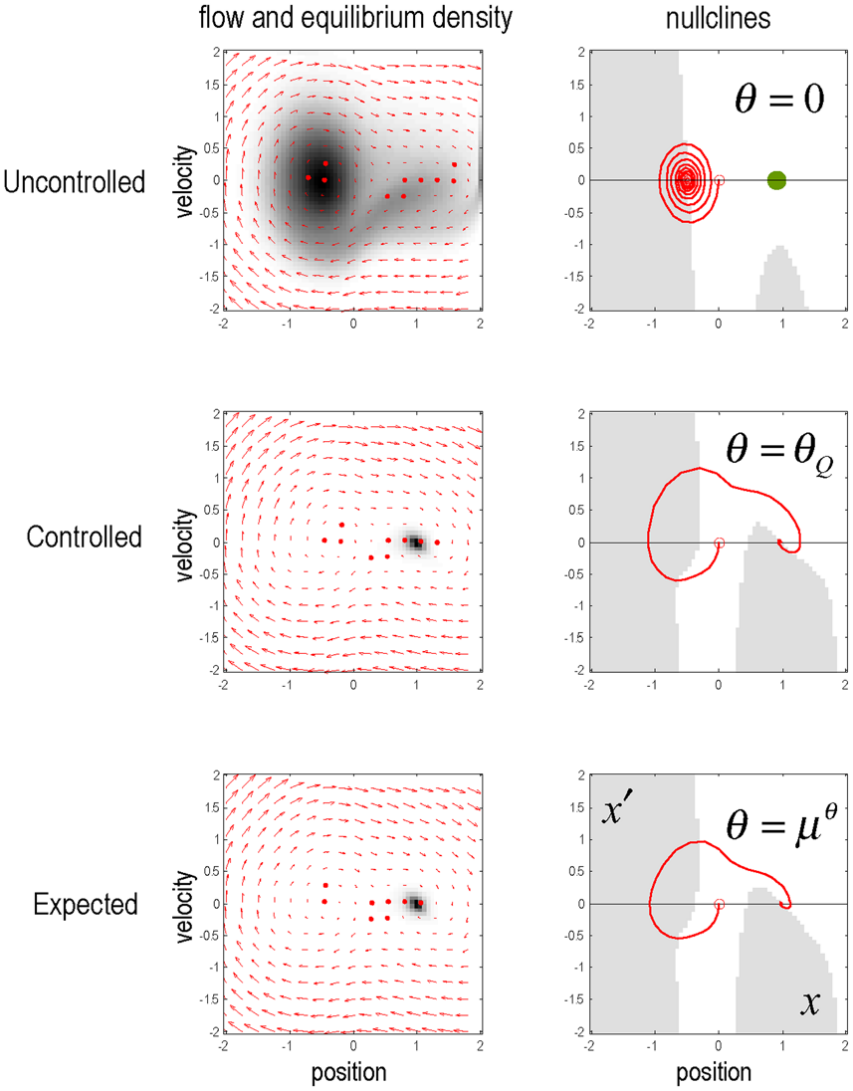
Equilibria in the state-space of the mountain car problem. Left panels: Flow-fields and associated equilibria for an uncontrolled environment (top), a controlled or optimised environment (middle) and under prior expectations after learning (bottom). Notice how the flow of states in the controlled environment enforces trajectories that start by moving away from the desired location (green dot at 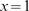). The arrows denote the flow of states (position and velocity) prescribed by the parameters. The equilibrium density in each row is the principal eigenfunction of the Fokker-Plank operator associated with the parameters. For the controlled and expected environments, these are low entropy equilibria, centred on the desired location. Right panels: These panels show the flow fields in terms of their nullclines. Nullclines correspond to lines in state-space where the rate of change or one variable is zero. Here the nullcline for position is along the x-axis, where velocity is zero. The nullcline for velocity is when the change in velocity goes from positive (grey) to negative (white). Fixed points correspond to the intersection of these nullclines. It can be seen that under an uncontrolled environment (top) there a stable fixed point, where the velocity nullcline intersects the position nullcline with negative slope. Under controlled (middle) and expected (bottom) dynamics there are three fixed points. The rightmost fixed-point is under the desired equilibrium density and is stable. The middle fixed-point is halfway up the hill and the final fixed-point is at the bottom. Both of these are unstable and repel trajectories so that they are ultimately attracted to the desired location. The red lines depict exemplar trajectories, under deterministic flow, from 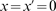. In a controlled environment, this shows the optimum behaviour of moving up the opposite hill to gain momentum so that the desired location can be reached.

The upper panels of Figure 3 show the equilibrium densities without control (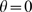; top row) and for the controlled environment that approximates our desired equilibrium (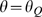; middle row). Here, 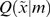 was a Gaussian density centred on *x* = 1 and 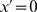 with standard deviation of 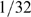
and 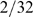 respectively. We have now created an environment in which the desired location attracts all trajectories. As anticipated, the trajectories in Figure 3 (middle row) move away from the desired location initially and then converge on it. This controlled environment now plays host to a naïve agent, who must learn its dynamics through experience.

### Learning a controlled environment

The agent's generative model of its sensory inputs comprised the functions
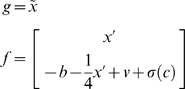
(15)


For simplicity, we assumed 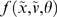 was the same as in Equation 13 but without action. The unknown causes in this model, 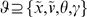, comprise the states (position and velocity), exogenous force, parameters controlling state-dependent acceleration and precisions (inverse variances) of the random fluctuations. Notice that the model has no notion of action; action is not part of inference, it simply tries to explain away any sensations that are not predicted. The agent was exposed to 16 trials of 32 second time-bins. Simulated training involved integrating Equation 12 with 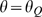. On each trial, the car was ‘pushed’ with an exogenous force, sampled from a Gaussian density with a standard deviation of eight. This enforced a limited exploration of state-space. The agent was aware of these perturbations, which entered as priors on the forcing term; i.e. 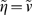 (see Equation 8). During learning, we precluded active inference, *a* = 0; such that the agent sensed its trajectory passively, as it was expelled from the desired state and returned to it.

Note that the agent does know the true states because we added a small amount of observation error (with a log-precision of eight) to form sensory inputs. Furthermore, the agent's model allows for random fluctuations on both position and velocity. When generating sensory data we used a small amount of noise on the motion of the velocity (log-precision of eight). After 16 trials the parameters converged roughly to the values used to construct the control environment. This means, in effect, the agent expects to be delivered, under state-dependent forces, to the desired state. These optimum dynamics have been learned in terms of (empirical) priors on the generalised motion of states encoded by 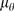, the expected parameters of the equations of motion. These expectations are shown in the lower row of Figure 3 in term of trajectories encoded by 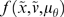. It can be seen that the nullclines (lower right) based on the parameters after training have a similar topology to the controlled environment (middle right), ensuring the fixed-points that have been learnt are the same as those desired. So what would happen if the agent was placed in an uncontrolled environment that did not conform to its expectations?

### Active inference

**Figure 4:**
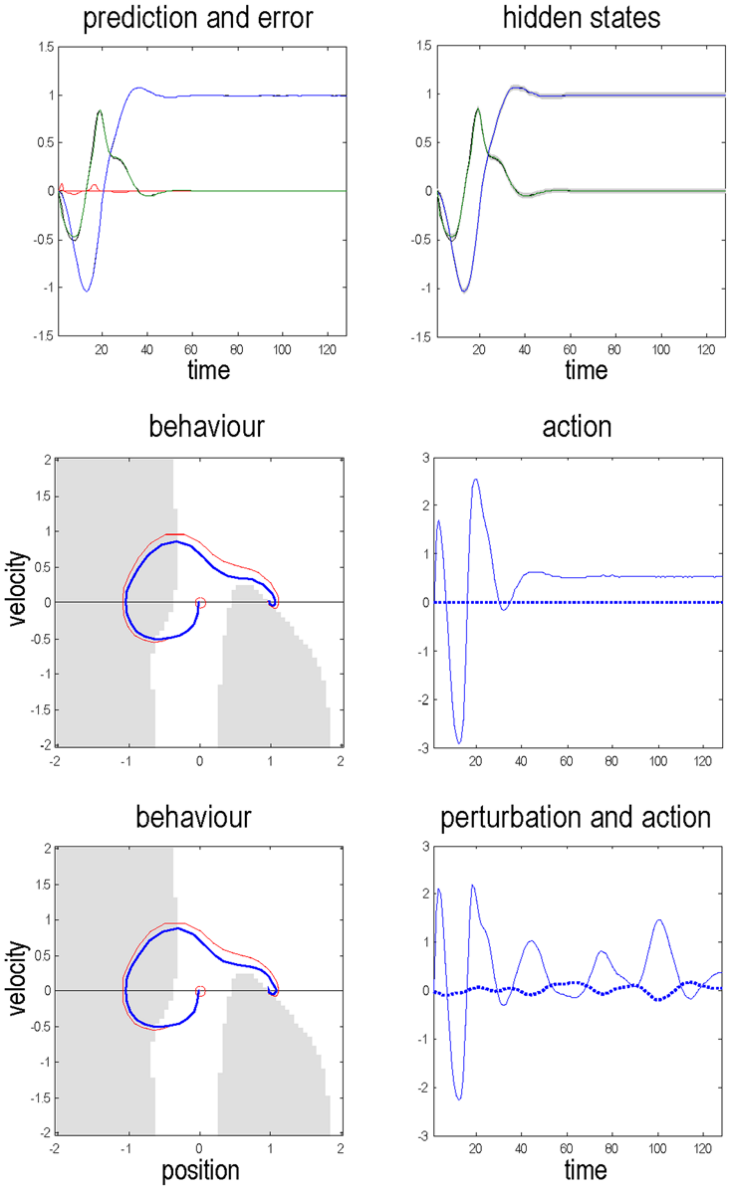
Inferred motion and action of an mountain car agent. Top row: The left panel shows the predicted sensory states (position in blue and velocity in green). The red lines correspond to the prediction error based upon conditional expectations of the states on (right panel). These expectations are optimised using Equation 9. This is a variational scheme that optimises the free-energy in generalised coordinates of motion. The associated conditional covariance is displayed as 90% confidence intervals (thin grey areas). Middle row: The nullclines and implicit fixed points associated with the parameters learnt by the agent, after exposure to a controlled environment (left). The actual trajectory through state-space is shown in blue (the red line is the equivalent trajectory under deterministic flow). The action causing this trajectory is shown on the right and shows a poly-phasic response, until the desired position is reached, after which a small amount of force is required to stop it sliding back down the hill (see Figure 2). Bottom row: As for the middle row but now in the context of a smoothly varying perturbation (broken line in the right panel). Note that this exogenous force has very little effect on behaviour because it is unexpected and countered by action. These simulations used expected log-precisions of: 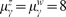.

To demonstrate the agent has learnt the optimum policy, we placed it in an uncontrolled environment; i.e., 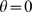 and allowed action to minimize free-energy. Although it would be interesting to see the agent adapt to the uncontrolled environment, we precluded any further perceptual learning. An example of active inference after learning is presented in Figure 4. Again this involved integrating environmental and recognition dynamics (Equations 7 and 9); where these stochastic differential equations are now coupled through action (Equation 12). The coloured lines show the conditional expectations of the states, while the grey areas represent 90% confidence intervals. These are very tight because we used low levels of noise. The dotted red line on the upper left corresponds to the prediction error; namely the discrepancy between the observed and predicted states. The ensuing trajectory is superimposed on the nullclines and shows the agent moving away from its goal initially; to build up the momentum required to ascend the hill. Once the goal has been attained action is still required because, in the test environment, it is not a fixed-point attractor.

To illustrate the robustness of this behaviour, we repeated the simulation using a smooth exogenous perturbation (e.g., a strong wind, modelled with a random normal variate, smoothed with a Gaussian kernel of eight seconds). Because the agent did not expect this, it was explained away by action and not perceived. The ensuing goal-directed behaviour was preserved under this perturbation (lower panels of Figure 4). Note the mirror symmetry between action and the displacing force it counters (action is greater because it exerts its effects through a squashing function).

In this example, we made things easy for the agent by giving it the true form of the process generating its sensory data. This meant the agent only had to learn the parameters. In a more general setting, agents have to learn both the form and parameters of their generative models. However, there is no fundamental distinction between learning the form and parameters of a model, because the form can be cast in terms of priors that switch parameters on or off (c.f., automatic relevance determination and model optimisation; [45]). In brief, this means that optimising the parameters (and hyperparameters) of a model can be used to optimise its form. Indeed, in statistics, Bayesian model selection is based upon a free-energy bound on the log-evidence for competing models [46]. They key thing here is that the free-energy principle reduces the problem of learning an optimum policy to the much simpler and well-studied problem of perceptual learning, without reference to action. Optimum control emerges when active inference is engaged.

### Optimal behaviour and conditional confidence

Optimal behaviour depends on the precision of expected motion of the hidden states encoded by 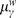. In this example, the agent was fairly confident about its prior expectations but did not discount sensory evidence completely (with log-precisions of 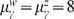). These conditional precisions are important quantities and control the relative influence of bottom-up sensory information relative to top-down predictions. In a perceptual setting they mediate attentional gain; c.f., [9], [47], [48]. In active inference, they also control whether an action is emitted or not (i.e., motor intention): Increasing the relative precision of empirical priors on motion causes more confident behaviour, whereas reducing it subverts action, because prior expectations are overwhelmed by sensory input and are therefore not expressed at the level of sensory predictions. In biological formulations of the free-energy principle, current thinking is that dopamine might encode the precision of prior expectations [39], [48]. A deficit in dopaminergic neurotransmission would reduce the operational potency of priors to elicit action and lead to motor poverty; as seen in Parkinson's disease, schizophrenia and neuroleptic bradykinesia.

**Figure 5:**
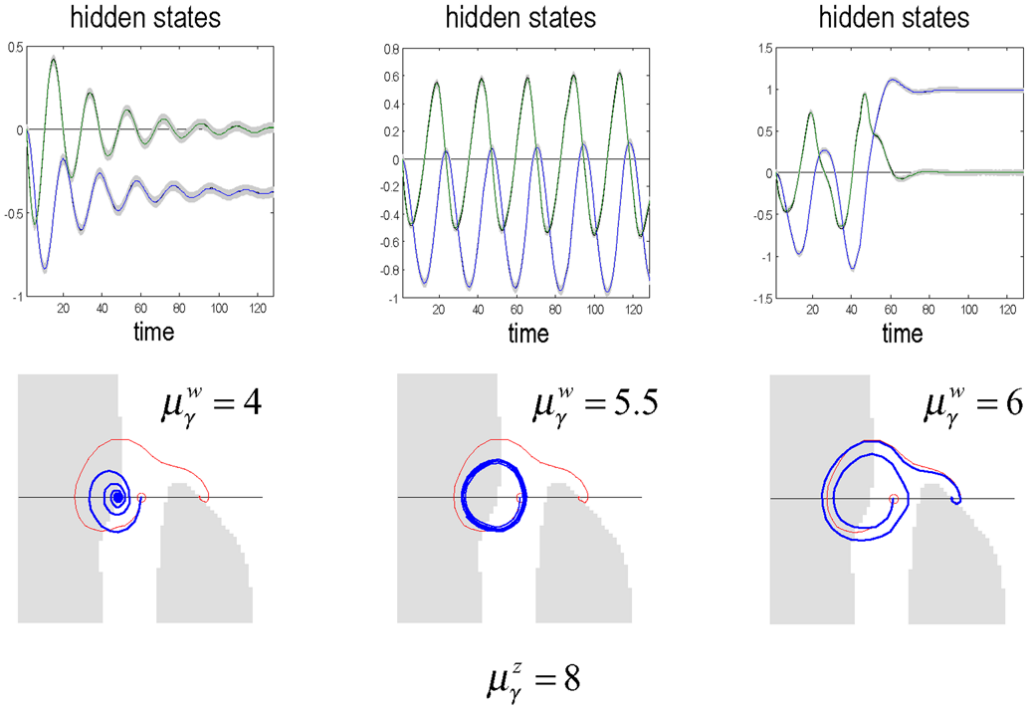
The effect of precision (dopamine) on behaviour. Inferred states (top row) and trajectories through state-space (bottom row) under different levels of conditional uncertainty or expected precision. As in previous figures, the inferred sensory states (position in blue and velocity in green) are shown with their 90% confidence intervals. And the trajectories are superimposed on nullclines. As the expected precision 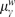 falls, the inferred dynamics are less accountable to prior expectations, which become less potent in generating prediction errors and action. It is interesting to see that uncertainty about the states (gray area) increases, as precision falls and confidence is lost.

By progressively reducing the expected precision of the empirical priors that have been instilled during training, we can simulate this poverty. Figure 5 shows three phases: first a loss of confident behaviour, where the car rocks itself backward and forward cautiously until it has more than sufficient momentum to reach its goal. Second, a stereotyped behaviour (corresponding to a quasi-periodic attractor), in which the car prevaricates at the bottom of the hill (c.f., displacement activity, motor stereotypy or perseveration). Finally, we get avolitional behaviour, where the car succumbs to gravity (c.f., bradykinesia or psychomotor poverty).

### Value and free-energy

So how does active inference relate to classical schemes? Dynamic programming and value-learning try to optimise a policy 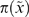 based on a value-function 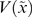 of hidden states, which corresponds to expected reward or negative cost. To see how this works, consider the optimal control problem



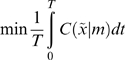
(16)Where, for infinite horizon problems, 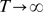 and 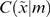 is some cost-function of hidden states that we want to minimise. The optimum control ensures the hidden states move up the gradients established by the value-function; i.e., action maximises value at every point in time
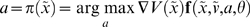
(17)


The value-function is the solution to the Hamilton Jacobi Bellman equation
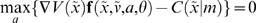
(18)


This equation comes from the theory of dynamic programming, pioneered in the 1950s by Richard Bellman and colleagues [2]. To optimise control 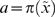 under this formulation, we have to: (i) assume the hidden states are available to the agent and (ii) solve Equation 18 for the value-function. Solving for the value-function is a non-trivial problem and usually involves backwards induction or some approximation scheme like reinforcement-learning [4]–[6]. The free-energy formulation circumvents these problems by prescribing the policy in terms of free-energy, which encodes optimal control (Equation 6)
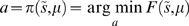
(19)


This control is specified in terms of prior expectations about the trajectory of hidden states causing sensory input and enables the policy to be specified in terms of sensory states 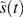 and expectations encoded by 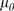. These expectations are induced by learning optimal trajectories in a controlled environment as above.

When constructing the controlled environment we can optimise the trajectories of hidden states without reference to policy optimisation. Furthermore, we do not have to consider the mapping between hidden and sensory states, because the controlled environment does not depend upon how the agent samples it. Optimal trajectories are specified by 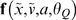, where 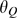 is given by Equation 14 and a desired density 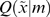. If we assume this density is a point mass at a desired state, 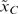


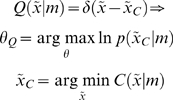
(20)


This means the optimal parameters maximise the probability of ending in a desired state, after a long period of time (i.e., under the equilibrium density).

Clearly, under controlled equilibria, 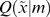 encodes an implicit cost-function but what about the uncontrolled setting, in which agents are just trying to minimise their sensory entropy? Comparison of Equations 17 and 19 suggests that value is simply negative free-energy; 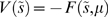. Here, value is been defined on sensory states, as opposed to hidden states. This means, valuable states are unsurprising and, by definition, are the sensory states available within the agent's environmental niche.

### Summary

In summary, the free-energy formulation dispenses with value-functions and prescribes optimal trajectories in terms of prior expectations. Active inference ensures these trajectories are followed, even under random perturbations. In what sense are priors optimal? They are optimal in the sense that they restrict the states of an agent to a small part of state-space. In this formulation, rewards do not attract trajectories; rewards are just sensory states that are visited frequently. If we want to change the behaviour of an agent in a social or experimental setting, we simply induce new (empirical) priors by exposing the agent to a new environment. From the engineering perceptive, the ensuing behaviour is remarkably robust to noise and limited only by the specification of the new (controlled) environment. From a neurobiological perceptive, this may call for a re-interpretation of the role of things like dopamine, which are usually thought to encode the prediction error of value [49]. However, dopamine may encode the precision of prediction errors on sensory states [39]. This may reconcile the role of dopamine in movement disorders (e.g., Parkinson's disease; [50]) and reinforcement learning [51], [52]. In brief, stimuli that elicit dopaminergic responses may signal that predictions are precise. These predictions may be proprioceptive and elicit behavioural responses through active inference. This may explain why salient stimuli, which elicit orienting responses, can excite dopamine activity even when they are not classical reward stimuli [53], [55]. Furthermore, it may explain why dopamine signals can be evoked by many different stimuli; in the sense that a prediction can be precise, irrespective of what is being predicted.

## Discussion

Using the free-energy principle, we have solved a benchmark problem in reinforcement learning using a handful of trials. We did not invoke any form of dynamic programming or value-function: Typically, in dynamic programming and related approaches in economics, one posits the existence of a value-function of every point in state-space. This is the reward expected under the current policy and is the solution to the relevant Bellman equation [2]. A policy is then optimised to ensure states of high value are visited with greater probability. In control theory, value acts as a guiding function by establishing gradients, which the agent can ascend [2], [3], [5]. Similarly, in discrete models, an optimum policy selects states with the highest value [4], [6]. Under the free-energy principle, there is no value-function or Bellman equation to solve. Does this mean the concepts of value, rewards and punishments are redundant? Not necessarily; the free-energy principle mandates action to fulfil expectations, which can be learned and therefore taught. To preclude specific behaviours (i.e., predictions) it is sufficient to ensure they are never learned. This can be assured by decreasing the expected precision of prediction errors by exposing the agent to surprising or unpredicted stimuli (i.e., punishments like foot-shocks). By the same token, classical rewards are necessarily predictable and portend a succession of familiar states (e.g. consummatory behaviour). It is interesting to note that classical rewards and punishments only have meaning when one agent teaches another; for example in social neuroscience or exchanges between an experimenter and subject. It should be noted that in value-learning and free-energy schemes there are no distinct rewards or punishments; every sensory signal has an expected cost, which, in the present context, is just surprise. From a neurobiological perspective [51]–[56], it may be that dopamine (encoding 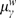) does not encode the *prediction error of value* but the *value of prediction error*; i.e., the precision of prediction errors that measure surprise to drive perception and action.

We claim to have solved the mountain car-problem without recourse to Bellman equations or dynamic programming. However, it could be said that we have done all the hard work in creating a controlled environment; in the sense that this specifies an optimum policy, given a desired equilibrium density (i.e., value-function of states). This may be true but the key point here is that the agent does not need to optimise a policy. In other words, it is us that have desired states in mind, not the agent. This means the notion that agents optimise their policy may be a category error, because the agent only needs to optimise its perceptual model. This argument becomes even more acute in an ecological setting, where there is no ‘desired’ density. The only desirable state is a state that the agent can frequent, where these states defines the nature of that agent.

In summary, we have shown how the free-energy principle can be harnessed to optimise policies usually addressed with reinforcement learning and related theories. We have provided proof-of-principle that behaviour can be optimised without recourse to utility or value functions. In ethological terms, the implicit shift is away from reinforcing desired behaviours and towards teaching agents the succession of sensory states that lead to desired outcomes. Underpinning this work is a unified approach to action and perception by making both accountable to the ensemble equilibria they engender. In the examples above, we have seen that perceptual learning and inference is necessary to induce prior expectations about how the sensorium unfolds. Action is engaged to resample the world to fulfil these expectations. This places perception and action in intimate relation and accounts for both with the same principle. Furthermore, this principle can be implemented in a simple and biologically plausible fashion. The same scheme used in this paper has been used to simulate a range of biological processes; ranging from perceptual categorisation of bird-song [57] to perceptual learning during the mismatch negativity paradigm [10]. If these ideas are valid; then they suggest that value-learning, reinforcement learning, dynamic programming and expected utility theory may be incomplete metaphors for how complex biological systems actually operate and speak to a fundamental role for perception in action; see [58]–[60] and [61].
